# Evaluation of high-throughput functional categorization of human disease genes

**DOI:** 10.1186/1471-2105-8-S3-S7

**Published:** 2007-05-09

**Authors:** James L Chen, Yang Liu, Lee T Sam, Jianrong Li, Yves A Lussier

**Affiliations:** 1 Department of Medicine, Georgetown University Hospital, Washington, DC, USA; 2 Section of Genetic Medicine, Department of Medicine, University of Chicago, Chicago, Illinois, USA; 3 Department of Biomedical Informatics, Columbia University, New York, NY, USA

## Abstract

**Background:**

Biological data that are well-organized by an ontology, such as Gene Ontology, enables high-throughput availability of the semantic web. It can also be used to facilitate high throughput classification of biomedical information. However, to our knowledge, no evaluation has been published on automating classifications of human diseases genes using Gene Ontology. In this study, we evaluate automated classifications of well-defined human disease genes using their Gene Ontology annotations and compared them to a gold standard. This gold standard was independently conceived by Valle's research group, and contains 923 human disease genes organized in 14 categories of protein function.

**Results:**

Two automated methods were applied to investigate the classification of human disease genes into independently pre-defined categories of protein function. One method used the structure of Gene Ontology by pre-selecting 74 Gene Ontology terms assigned to 11 protein function categories. The second method was based on the similarity of human disease genes clustered according to the information-theoretic distance of their Gene Ontology annotations. Compared to the categorization of human disease genes found in the gold standard, our automated methods can achieve an overall 56% and 47% precision with 62% and 71% recall respectively. However, approximately 15% of the studied human disease genes remain without GO annotations.

**Conclusion:**

Automated methods can recapitulate a significant portion of classification of the human disease genes. The method using information-theoretic distance performs slightly better on the precision with some loss in recall. For some protein function categories, such as 'hormone' and 'transcription factor', the automated methods perform particularly well, achieving precision and recall levels above 75%. In summary, this study demonstrates that for semantic webs, methods to automatically classify or analyze a majority of human disease genes require significant progress in both the Gene Ontology annotations and particularly in the utilization of these annotations.

## Background

The rapidly increasing volume of genomic data derived from high throughput technologies has made the analysis of human disease genes one of the primary challenges in clinical bioinformatics. The Semantic Web plays a key role in facilitating the fusion and dissemination of data these high-throughput methods generate. By working to integrate the heterogeneous types of data that result, the Semantic Web also has a role in maximizing the value of incumbent methods and technologies. Classification and clustering are some of the most common methods for organizing and providing descriptive statistics over a dataset and is expected to be widely used under the "semantic web framework". In terms of human diseases, this process may be valuable in discovering generalized principles of the relationship between human diseases and the molecular or biological mechanisms associated to their genes.

Well-structured data representation, such as genes annotated in Gene Ontology (**GO**), can enable automated high-throughput availability through the Semantic Web. However, to our knowledge, no studies have compared GO classification capabilities with that of human disease genes datasets independently annotated in molecular functions. To achieve this, Valle's research group manually classified nearly 1000 human disease genes found in the Online Mendelian Inheritance in Man according to protein product [1]. They found significant correlations between function and clinical disease phenotypes such as in age of onset, frequency, and mode of inheritance.

### Information theory

The basis of Information Theory is relevant to understanding the hypotheses and methods of this paper. Information theory was conceived by Claude Shannon at Bell Labs and published in 1948 [2]. At its heart is the definition of information content which is shown in Equation 1 for a message *m*, and its probability *p(m)*:

*I*(*m*) = -log *p*(*m*)     (Equation 1)

As the probability that a message will occur diminishes, its information content increases. In other words, an infrequently occurring message holds higher value. The probability of a concept occurring in an ontology is calculated in Equation 2 [3].

p(x)=(1+number of all descendants of x )total number of concepts in the ontology     (Equation 2)
 MathType@MTEF@5@5@+=feaafiart1ev1aaatCvAUfKttLearuWrP9MDH5MBPbIqV92AaeXatLxBI9gBaebbnrfifHhDYfgasaacH8akY=wiFfYdH8Gipec8Eeeu0xXdbba9frFj0=OqFfea0dXdd9vqai=hGuQ8kuc9pgc9s8qqaq=dirpe0xb9q8qiLsFr0=vr0=vr0dc8meaabaqaciaacaGaaeqabaqabeGadaaakeaacqWGWbaCcqGGOaakcqWG4baEcqGGPaqkcqGH9aqpdaWcaaqaaiabcIcaOiabigdaXiabgUcaRiabb6gaUjabbwha1jabb2gaTjabbkgaIjabbwgaLjabbkhaYjabbccaGiabb+gaVjabbAgaMjabbccaGiabbggaHjabbYgaSjabbYgaSjabbccaGiabbsgaKjabbwgaLjabbohaZjabbogaJjabbwgaLjabb6gaUjabbsgaKjabbggaHjabb6gaUjabbsha0jabbohaZjabbccaGiabb+gaVjabbAgaMjabbccaGiabdIha4jabbccaGiabbMcaPaqaaiabbsha0jabb+gaVjabbsha0jabbggaHjabbYgaSjabbccaGiabb6gaUjabbwha1jabb2gaTjabbkgaIjabbwgaLjabbkhaYjabbccaGiabb+gaVjabbAgaMjabbccaGiabbogaJjabb+gaVjabb6gaUjabbogaJjabbwgaLjabbchaWjabbsha0jabbohaZjabbccaGiabbMgaPjabb6gaUjabbccaGiabbsha0jabbIgaOjabbwgaLjabbccaGiabb+gaVjabb6gaUjabbsha0jabb+gaVjabbYgaSjabb+gaVjabbEgaNjabbMha5baacaWLjaGaaCzcaiabbIcaOiabbweafjabbghaXjabbwha1jabbggaHjabbsha0jabbMgaPjabb+gaVjabb6gaUjabbccaGiabbkdaYiabbMcaPaaa@9F7E@

The measure of semantic similarity between two concepts in a taxonomy was first proposed by Resnik [4] and later modified by Lin [5] to use the shared information content between two concepts as well as the information content of individual concepts to calculate semantic similarity. Taxonomies can essentially be regarded as simplified ontologies in which the only type of relations between concepts is the IS-A relation. Consequently, these algorithms can be applied without modification. Resnik defines semantic distance in terms of the minimum subsumer of concepts *a *and *b *as shown in Equation 3. The minimum subsumer is defined as the common ancestor concept between *c*_*i *_and *c*_*j *_that has the minimal probability of occurrence or the minimal number of descendants.

*sim*(*c_i_*, *c_j_*) = -log(*p*(*ms*(*c_i_*, *c_j_*)))     (Equation 3)

The major drawback of this formula is in its consideration of only the shared information content between two concepts, but ignoring the information content of each individual concept. Lin uses this information in a more complex calculation shown in Equation 4. This approach normalizes the information content between two concepts to a range between 0 and 1 using the two concepts' information content.

sim(ci,cj)=2×max⁡[−log⁡(p(c))]log⁡(p(ci)+log⁡(p(cj))  where  c∈S(ci,cj)     (Equation 4)
 MathType@MTEF@5@5@+=feaafiart1ev1aaatCvAUfKttLearuWrP9MDH5MBPbIqV92AaeXatLxBI9gBaebbnrfifHhDYfgasaacH8akY=wiFfYdH8Gipec8Eeeu0xXdbba9frFj0=OqFfea0dXdd9vqai=hGuQ8kuc9pgc9s8qqaq=dirpe0xb9q8qiLsFr0=vr0=vr0dc8meaabaqaciaacaGaaeqabaqabeGadaaakeaacqWGZbWCcqWGPbqAcqWGTbqBcqGGOaakcqWGJbWylmaaBaaameaacqWGPbqAaeqaaOGaeiilaWIaem4yam2cdaWgaaadbaGaemOAaOgabeaakiabcMcaPiabg2da9maalaaabaGaeGOmaiJaey41aqRagiyBa0MaeiyyaeMaeiiEaGNaei4waSLaeyOeI0IagiiBaWMaei4Ba8Maei4zaCMaeiikaGIaemiCaaNaeiikaGIaem4yamMaeiykaKIaeiykaKIaeiyxa0fabaGagiiBaWMaei4Ba8Maei4zaCMaeiikaGIaemiCaaNaeiikaGIaem4yam2cdaWgaaadbaGaemyAaKgabeaakiabcMcaPiabgUcaRiGbcYgaSjabc+gaVjabcEgaNjabcIcaOiabdchaWjabcIcaOiabdogaJTWaaSbaaWqaaiabdQgaQbqabaGccqGGPaqkcqGGPaqkaaGaaGPaVlaaykW7cqqG3bWDcqqGObaAcqqGLbqzcqqGYbGCcqqGLbqzcqqGGaaicqqGGaaitCvAUfeBSjuyZL2yd9gzLbvyNv2CaeHbuLwBLnhiov2DGi1BTfMBaGabciaa=ngacqGHiiIZcaWFtbGaeiikaGIaem4yam2cdaWgaaadbaGaemyAaKgabeaakiabcYcaSiabdogaJTWaaSbaaWqaaiabdQgaQbqabaGccqGGPaqkcaWLjaGaaCzcaiabbIcaOiabbweafjabbghaXjabbwha1jabbggaHjabbsha0jabbMgaPjabb+gaVjabb6gaUjabbccaGiabbsda0iabbMcaPaaa@9AB1@

The equation states that S(c_i_, c_j_) represents the parent terms shared by both c_i _and c_j_. 'Max' represents the maximum operator.

Methods based on Shannon's information theory have been applied in a number of methods for clustering genes by functional annotations. Wang, et al. applied an information theoretic semantic similarity measure to measure similarity between gene products [6]. Similarly, Steuer, et al. used a mutual information model to evaluate gene cluster membership based on GO classes [7].

### Hypothesis

We developed a set of hypotheses surrounding the use of existing databases and semantic techniques to recapitulate the sets derived from the manual categorization effort of Valle's research group in 2001. Our primary hypothesis is based on the assumption that we may be able to classify diseases using the structure of Gene Ontology. We hypothesized that a manual selection of GO classes homologous to those of Valle's categories of protein functions would recapitulate Valle classification of genes. We further hypothesized that we may be able to derive 'natural functional categories' through the application of semantic relationships between human disease genes within the Gene Ontology deriving from more GO annotations than those that were homologous to Valle's categories. This global classification approach implemented using information theory and clustering may be superior at capturing subtle functional similarities than through direct use of the ontology's structure. This experiment aims to compare the accuracy of the two hypothesized classification methods against a gold standard set of human disease genes that was organized independently of GO (Valle's dataset) [1]. While ontology-anchored classification methods abound, very few studies provide a formal evaluations of their accuracy using independently annotated datasets and, more importantly, independently conceived protein function classifications.

## Results

### Experimental design

In their paper, the Valle research group classified human disease genes (HDG) by disease frequency, mode of inheritance, and protein product function. We chose to concentrate our efforts on the last category, protein function. Of the original 923 human disease genes that Valle's group classified, we examined the 787 with mappings into GO. For the first classification method (GO-mapping), we first mapped 12 out of the 14 protein function categories in their study to the most relevant terms from GO (Table 1). Protein function categories 'others' and 'unknown" were not mapped due to their ambiguity, and two categories pertaining to 'transport' were merged as current GO terms did not have draw this distinction. This process resulted in a set of 72 distinct GO terms covering 12 of Valle's categories of protein function. In the second method (GO-clustering), we classified the disease genes by calculating the information-theoretic distances between their associated GO terms [6]. Based on these similarity scores, we then clustered the human disease genes into 14 classes by repeated-bisection clusters (details in the Methods). Precision and recall were calculated by comparison against Valle's original categories and gene annotations.

### Results of the GO mapping method to classify HDGs

We selected 72 GO terms that best mapped to 12 Valle's functional classifications. Of a total 923 human disease genes (HDG), 136 were excluded due to their lack of mappings into the GO database based on their associated LocusLink IDs. From the set of 787 disease genes that passed this filter, 728 (92.6%) were successfully assigned to the 72 selected GO terms, and were then mapped to Valle's classification (Table 2). 59 entries (7.4%) were not assigned to any of the selected GO terms. In some categories, the mapping performed well for many protein functions as shown in Table 2. For example, the 'hormone' class achieved 93% precision and 100% recall; and the 'transcription factor' class achieved 85% precision and 78% recall. However, some classes such as 'intracellular matrix component' reached only 10% precision and 46% recall. The total recall is 71% and total precision is 47%.

Due to the ontological structure of GO, human disease genes could be assigned to several GO terms. Of the 728 HDG, 279 had one mapping, 348 had two mappings, 84 had three mappings, and 17 had four mappings (as shown in Figure 2). In some cases, GO terms define the HDG more specifically than Valle's original classification, which only consider one function of a gene. For example, of the 17 HDG with four mappings, four belong to cholinergic receptors (OMIM:100690, OMIM:100710, OMIM:100725, OMIM:118504). Based on the OMIM biochemical description, these acetylcholine receptors are transmembrane proteins that act as pores that create a tapering path for ions to enter the cell. In the original classification, these HDGs were tagged as receptors. As noted in Table 2, we calculated recall for each category in isolation. Due to the mapping of a number of genes to multiple GO categories, they could also be categorized in more than one protein function could be counted as false positive and true positive. This inflated the overall number of false positives estimated by this method, leading to a conservative calculation of precision. In "classical" evaluations, a gene assignment is counted only once as either true positive, false positive or false negative – however these classical accuracy metrics are not well suited to determine accuracies for methods allowing multiple categorizations of one gene.

### Results of the GO Clustering method to classify HDGs

We took 787 human disease genes from Valle's dataset with GO annotations and automatically classified them according to similarities of their information theoretic distances in GO (details are described in the Methods). As shown in Figure 2, the majority of proteins had between 6 and 12 GO annotations. 4,722 distinct GO annotations were found. In this GO-Clustering method, every exact GO annotation was kept associated to the gene. Each GO annotation had fewer associated genes than in the previous method where genes were lumped into 72 GO mappings. Genes associated with GO terms that were descendent of the original 74 groups are subsumed in each of these groups, increasing the number of genes "*indirectly*" associated with each of the 74 GO terms. This analysis took the greatest amount of time in calculation of the information-theoretic distance. Each HDG code had on average 6 associated GO annotations. Therefore there were 787 × (787-1)/2 comparisons using 4,722 GO terms × 4,722 GO terms, which resulted in approximately 22 million pair-wise information-theoretic calculations. Repeated-bisection clustering was applied to divide the 787 HDG into 14 clusters, which were then mapped to Valle's functional classifications [8]. Table 3 shows the comparison of the classification using GO-clustering methods with Valle's classification. In terms of recall, the 'hormone' and 'channel' clusters were best at recapitulating the gold standard in both top clusters (recall 79%, 88%) and top two clusters (recall 93%, 100%). 'Enzyme' and 'transcription factor' were ranked highest in terms of precision (81%, 80%, respectively) which also held when the top two categories were combined (89%, 59%, respectively). The total recall is 62% with 56% precision. The detailed mapping of the 14 clusters using Valle's classifications is shown in Table 4. Assignment of a cluster to one of Valle's protein function categories was based on the larger absolute value of TP scores for that category as shown in Table 4. Obviously, no cluster was found to be associated with the ambiguous "other" and "unknown" protein function categories. As noted earlier for the GO Mapping technique, the Gene Ontology classification does not provide sufficient granularity to differentiate between the two gold standard categories of "transport". This is also reflected in the results of the GO Clustering method, as no cluster matches "EC transport" (Column 8, Table 4).

## Discussion

### Comparison of GO mapping and GO clustering methods

**Figure 1:**
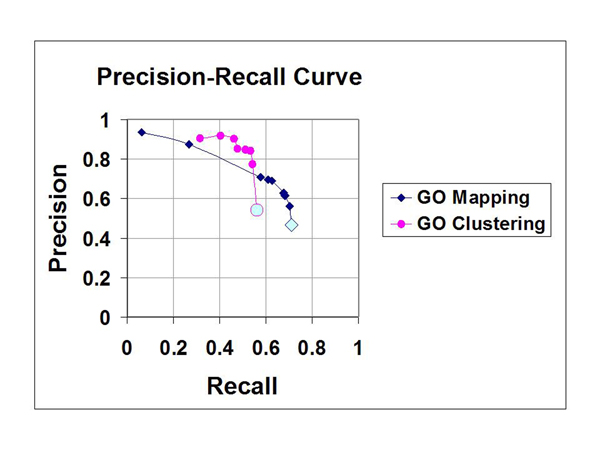
**Precision-recall graph for comparing accuracies of GO Mapping and GO Clustering methods**. The data points represented by a larger circle and square with empty centers correspond to the overall accuracy scores of the two methods. Additional points on the precision-recall curve were obtained by progressively removing classes with poor precision from the evaluated set. Vale's Human Disease Gene annotations was used as a Gold Standard to calculate precision and recall, the task being to recapitulate Valle's categorization of human disease genes via GO Mapping or GO Clustering as described in the methods. Note that between 30%–55% recall, the GO Clustering Method provides higher precision than the GO Mapping. Overall, GO Mapping provides about 15% higher recall, but 10% lower precision than GO Clustering.

**Figure 2:**
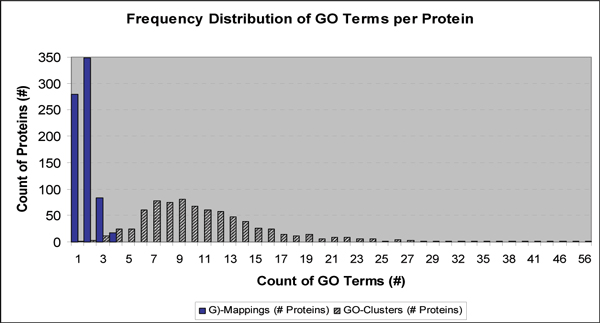
**Frequency distribution of the proteins associated to GO annotations**. Note that in order to construct the GO mappings, all genes in GO terms subsumed by each of the 74 selected GO classes in the GO ontology were aggregated in this higher level class, thus accounting for the large number of GO terms per class in GO mappings. In contrast, the GO-Cluster method retains the original granularity of the GO mappings and allows for GO terms not subsumed with the selected 74 GO classes selected for GO Mappings.

As shown in Figure 2, the two automated categorization methods are based on very different distributions of GO-gene annotations. The GO-mapping technique comprises relatively few GO classes to which a large number of genes are classified, while the GO-clustering method allows for the retention of a large number of distinct GO annotations and their mappings. When examining overall performance using Valle's classification as a gold standard, classification using the GO mapping method has an overall recall 71%, compared with 62% in the GO clustering method. However, the GO mapping method generates 47% precision and the GO clustering method generates 56% precision. Overall, GO-mapping provides about 15% higher recall than the GO-clustering method, but sacrifices about 10% precision. As higher precision is generally considered a more difficult task – it seems that taking into account all GO annotations rather than a subset may justify the computationally intensive GO-clustering method proposed by Wang and Bodenreider [6]. The precision-recall curve (Figure 1) illustrates the region from 35% recall to 55% recall where the precision of GO-clustering outperforms the GO-mapping method.

### Limitations of the GO-term selection method and an argument for larger expressiveness

The generation of functional views of proteins hinges on three key factors:

1) *GO Content*: the presence of GO annotations for the Human Disease Gene of interest,

2) *Ambiguity of data representation*: a sharply-outlined definition of the function of interest

3) *Expressiveness of the relationship model*: an understanding of the representation models of data and ontological class being selected

First, in this experiment, we found that about 15% of HDGs were not annotated in GO and we have not accounted for these genes in the overall calculations of accuracies. Taking into account these genes would have further lowered the recall of both categories, but the precision would have remained the same.

Second, some categories (e.g. 'modulator of protein') performed surprisingly poorly in terms of precision and recall in the GO Mapping method (precision 44%, recall 4%). This can be attributed to the ambiguity of the categorization task. Without looking at OMIM, 'modulator of protein function' may be described as *chaperone proteins *or as *enzymatic cofactors*. The inclusion of coagulation cascade factors was also not included in our original formulation. Indeed, a Pubmed search attempting to relate 'protein modulator' or 'modulator or protein function' with the term 'fibrinogen' does not yield any results.

Third, the proper initial selection of the GO term is also essential when a gene is allowed to have multiple classes in GO to a strict dichotomic classification as the one proposed by Valle's group. There is a clash of representational models between GO, a directed acyclic graph, and a dichotomic categorization task. While ontologies have seen increasing use, there is a pressing need to improve statistical manipulation of data categorized in multiple classes. The categorization task is somewhat artificial and accuracy scores do not reflect of the richness of alternate data models that can be produced by GO. This is due, by design, to our choice of a strict dichotomic categorization for a gold standard. In Valle's original classification, the authors stated that when there are more than one possible class, they would pick the *most defining category*. However, it is difficult to automate this defining categorization when a gene has multiple features in GO. For example, all 'channels' proteins were also found to be annotated as 'transmembrane proteins' in GO, but a protein was assigned to one and only one of these two categories in Valle's set. When is 'channel' the more appropriate class then 'transmembrane transporter'? Therefore, it is sometimes arbitrary to assign just one function classification to a gene.

### Limitations of information-theoretic distance metrics

The information-theoretic distance between two human diseases relies on the existence of sufficient annotation with ontological terms and sufficient depth of the term for a significant correlation. In other words, a shallow ontology or shallow labeling may provide enough discrimination. Ontological structure aside, the "Euclidian average of information-theoretic distance" calculation (Equation 5, Methods) which we used has two obvious disadvantages. First, our calculations may be biased by through comparison of distant concepts. For example, let us say that disease 1 has GO annotations A and B and disease 2 has annotations C and D. Let us say that "A and C" as well as "B and D" are similar concepts, while "A and D" and "B and C" are in different branches of the ontology entirely and thus very dissimilar. An average similarity uses every combination: AC, AD, BC and BD and would thus add very close associations with distant associations which when averaged ultimately result in a moderate association. It is striking to observe that two diseases sharing two GO annotations which taken one at a time each have a very similar homologous GO annotation in the other diseases would average a mediocre overall similarity score. We are exploring an improved distance metric between groups of GO terms which would control for the previously described bias. One solution is to compare each GO annotation of one disease to its most similar one in the other disease rather than to all annotations.

### Limitations of the clustering method

The choice of cluster count was arbitrarily set to the same number of categories in the Gold Standard, albeit one class was named 'Other' and another class 'unclassified'. A more rigorous analysis should be undertaken to optimize cluster size. One can speculate that as we decrease the number of clusters, the categories with members that were divided among several clusters would rejoin. As we saw, for a large category like 'enzyme', simply taking the two clusters instead of one doubled its recall without affecting the precision. Eventually, however, we would most likely see a corresponding decrease in precision. We are currently investigating kernel-based self-organized maps that may help adjust to the data in a more appropriate manner. In addition, it is important to mention that in contrast to the GO-Mapping method that directly categorizes into Valle's protein function categories, clustering does not attribute a name to a cluster. In practice, if this method were used over large datasets, results of each cluster would be sampled in order to estimate a precision and recall score according to the targeted categorization task at hand. Thus for a categorization task known *a priori*, the GO-Clustering method is more demanding both computationally and also in terms of knowledge engineering/evaluation. However, if one is interested in naturally clustering categories and to identify their meaning *a posteriori*, this technique is less demanding in knowledge engineering or evaluation than the GO Mapping one.

### Relationship of precision to recall

In information retrieval literature, it is well established that there should be an inverse relationship between precision and recall. As recall improves, precision should decrease. In other words, the more comprehensive the functional category is, the more likely it will also contain irrelevant diseases. Our data, in particular examining the GO-term mapping method and the top two GO-cluster points, reveal more of a positive correlation.

### Future studies

We intend to incorporate additional phenotypic ontology annotation of genes (e.g. annotations to Cell Ontology) in order to generate more accurate classifications that take into account cellular-specific expression of genes.

## Conclusion

Our automated methods can recapitulate a significant portion of classification of the human disease genes. The method using information theoretic distance performs slightly better on the precision with some loss in recall. Though in some categories, high precision and recall are reached, there are many issues that cause the final precision and recall not at the higher level. The performance of the GO-mapping method and the GO-clustering method is relatively similar even with their intrinsic differences. In addition, the differences in representational models made the use of traditional accuracy methods difficult to implement and interpret: GO is a directed acyclic graph allowing multiple parents, while clusters and categories are exclusive hierarchies allowing for only one category per gene. Thus, the precision of the straightforward GO-mapping method is underestimated because a gene can be counted twice, thus underestimating precision and possibly overestimating recall. In summary, this study demonstrates that for Semantic Web methods to automatically classify or analyze a majority of human disease genes, significant progress is required in several areas: (i) *content *and *disambiguation *in Gene Ontology annotations to allow for more extensive and less ambiguous mapping of human disease genes (85% of which could be mapped in GO), and (ii) *tolerance of the expressiveness of the GO representation model*. Indeed, the stringent traditional accuracy metrics did not gracefully account for the expressiveness of GO annotations. Improvements in the utilization of these annotations for categorization of genes are needed – perhaps allowing for multiple categorization as well as better evaluation metrics to compare *exclusive categorization *methods to *multihierarchic classification *methods, such as GO, that allow for a gene to be classified in more than one category.

## Methods

### Datasets

In this study, we used the following 4 datasets. 1) Gene Ontology Annotation (GOA) (downloaded January 2005) [9,10]. 2) OMIM to LocusLink mapping from the NCBI ftp server . mim2loc.txt.gz (downloaded August 2004). 3) LocusLink to GO mapping from the NCBI ftp server  loc2go.txt.gz (downloaded August 2004). 4) Human disease genes [1] listings and annotations are from the supplementary information site [11]. This dataset contains mappings of human diseases to their genetically corresponding functional categories.

### Classification of HDG using GO categories

(Refer to Table 1: GO terms mapped to Protein Function Categories)

For each gold standard functional category, we wanted to create a GO equivalent category. Therefore, we selected a set of corresponding GO terms (74 out of 2030 distinct GO terms and all descendents of these 74 GO mappings in the GO ontology) in all three GO ontologies: biological process, molecular function, and cellular component. Mappings were made based on semantic match or on the original purpose of the class. For example, the functional category of 'receptor' was mapped to receptor activity (GO:0004872), receptor binding (GO:0005102), cell surface receptor linked signal transduction (GO:0007166), and receptor signaling protein activity (GO:0005057).

We faced a number of challenges during the mapping process. For example, there is no distinction in GO between extracellular membrane (ECM) transporter and transmembrane transporter. Consequently, these classes were merged into the encompassing category transporter, which includes both the ECM transporter and transmembrane transporter classes. In addition, there was considerable overlap between functional classifications. The most extreme example was the class 'channel'. 'Channel' in GO is a subclass of 'transporter'. Thus Every HDG classified as a 'channel' was also classified as 'transporter'.

From the original 923 human disease gene subset of OMIM, 787 of the HDGs had been mapped to entries in GO based on a composite of the MIM to LocusLink and LocusLink to GO tables. In order to analyze the manual mappings, we iterated through each HDG. For each HDG, all corresponding GO terms, including respective superclasses, were aggregated. If a GO term matched a GO term previously identified as characteristic of a functional category in the gold standard, the MIM term was tagged with that category. The number of HDG in each of the new categorizations then compared to the gold standard categories through descriptive statistics.

### Automated clustering of human diseases using their information theoretic distance in GO

As in the previous mapping case, we generated a denormalized table containing each of the available HDG and their corresponding GO terms. From here, each HDG was compared pair-wise to all available HDG according to their mapped GO terms. Similarity and distance values for each pair of disease genes was calculated based on the information-theoretic distance developed first by Lin in 1998 [5] to evaluate sets of ontological terms (**Equation 4**, Background). It was then further refined by Wang et al [6] based on work by Cao et al [12] extending the information-theoretic distance to encompass gene-to-gene comparisons where each gene is annotated with multiple GO terms. Here, we used the normalized version of the information-theoretic model that states that similarity of two terms (GO annotations) *c*_*i*_, *c*_*j *_(**Equation 4**, background).

For our HDG pair-wise comparison, an information-theoretic score was computed. The scores were then aggregated and a Euclidian Average of Information Theoretic Distance between the HDG pair calculated. So for a given two HDG, *h*_1 _and *h*_2 _with corresponding set of GO terms *A*_*i*_*, A*_*j *_respectively (and terms *a *and *b *indicating the number of members in each of the sets) we define the average interest similarity as:

sim(h1,h2)=1a×b×∑cm∈Ai,cn∈Ajsim(cm,cn)     (Equation 5)
 MathType@MTEF@5@5@+=feaafiart1ev1aaatCvAUfKttLearuWrP9MDH5MBPbIqV92AaeXatLxBI9gBaebbnrfifHhDYfgasaacH8akY=wiFfYdH8Gipec8Eeeu0xXdbba9frFj0=OqFfea0dXdd9vqai=hGuQ8kuc9pgc9s8qqaq=dirpe0xb9q8qiLsFr0=vr0=vr0dc8meaabaqaciaacaGaaeqabaqabeGadaaakeaacqWGZbWCcqWGPbqAcqWGTbqBcqGGOaakcqWGObaAlmaaBaaameaacqaIXaqmaeqaaOGaeiilaWIaemiAaG2cdaWgaaadbaGaeGOmaidabeaakiabcMcaPiabg2da9maalaaabaGaeGymaedabaGaemyyaeMaey41aqRaemOyaigaaiabgEna0oaaqafabaGaem4CamNaemyAaKMaemyBa0MaeiikaGIaem4yam2cdaWgaaadbaGaemyBa0gabeaakiabcYcaSiabdogaJTWaaSbaaWqaaiabd6gaUbqabaGccqGGPaqkaSqaaOGaem4yam2cdaWgaaadbaGaemyBa0gabeaakiabgIGiolabdgeabTWaaSbaaWqaaiabdMgaPbqabaGccqGGSaalcqWGJbWylmaaBaaameaacqWGUbGBaeqaaOGaeyicI4Saemyqae0cdaWgaaadbaGaemOAaOgabeaaaSqab0GaeyyeIuoakiaaxMaacaWLjaGaeeikaGIaeeyrauKaeeyCaeNaeeyDauNaeeyyaeMaeeiDaqNaeeyAaKMaee4Ba8MaeeOBa4MaeeiiaaIaeeynauJaeeykaKcaaa@6EC4@

For example, acute promyelocytic leukemia (OMIM:102578) was compared to oncogene GLI3 (OMIM:165240). Acute promyelocytic leukemia has 10 GO labels including ubiquitin ligase complex (GO:0000151) and DNA-dependent regulation of transcription (GO:0006355). Oncogene GLI3 has 10 GO labels as well including DNA-dependent regulation of transcription (GO:0006355) and zinc ion binding (GO:0008270). Therefore, 10 GO annotations were compared from the first HDG to 10 GO annotations from the second HDG. The similarity then was calculated to be 0.74 – which indicates high correlation. This again, would be expected given that they are both oncogenes. The resulting correlation table was subsequently clustered through a self-organized map using a repeated-bisection algorithm in the CLUTO [11].

### Clustering methodology

Fourteen clusters were chosen to correspond to the original fourteen classifications in the original paper: unknown, enzyme, transcription factor, receptor, hormone, channel, transmembrane transporter, extracellular transporter, modulator of protein function, other function, extracellular matrix component, intracellular matrix component, immunoglobulin, and cell signaling. We used CLUTO to implement a *k*-way clustering solution in which, the entire collection of HDG-HDG correlations was first divided in half. Then one of the clusters is selected and is further bisected leading to three clusters. This process of cluster selection and bisection continues until *k *clusters are obtained [13].

### Evaluation

The annotation of 923 Human Disease Genes in 14 categories of protein function published by Valle's research group served as a gold standard [1]. The terms used in the evaluation are defined here. True positives (TP) are defined as instances where there was the same classification in both the gold standard and the GO-term method. False positives (FP) were defined as instances where the HDG placed in a functional category but not found in the gold standard. False negatives (FN) were instances where HDG were classes found in the gold standard to be in a category but not labeled in any instance to be in that category. The precision and recall for each category was then measured as TP/(TP+FP), and TP/(TP+FN), respectively. Each calculation for precision and recall per category were considered independent of one another. Therefore, one HDG could be counted as the TP for two categories.

### Software

All scripts were written in PERL or SQL. Repeated-bisection clustering performed by publicly available CLUTO software [13].

## Competing interests

The authors declare that they have no competing interests.

## Authors' contributions

Every author contributed to the redaction of the manuscript and the conceptualization of some of the methods. Contribution to the computations and analyzes from the most contributions to the least: James Chen, Jianrong Li, Yang Liu and Yves A. Lussier. The background and review of literature were mainly contributed by Lee Sam and James Chen. The interpretation and discussion was share between Yves Lusier, Yang Liu and James Chen. The overall supervision was conducted by Yves Lussier.

## Figures and Tables

**Table 1:** GO terms mapped to Valle's HDG functional categories

**Valle's functional classifications**	**GO terms**	**GO names**
Enzyme	GO:0050662	mf:coenzyme binding
Enzyme	GO:0019899	mf:enzyme binding
Enzyme	GO:0050790	bp:regulation of enzyme activity
Enzyme	GO:0003824	mf: catalytic activity
Enzyme	GO:0016591	cc: DNA-directed RNA polymerase II, holoenzyme
Enzyme	GO:0005697	cc: telomerase holoenzyme complex
Enzyme	GO:0017101	cc: aminoacyl-tRNA synthetase multienzyme complex

Modulator protein function	GO:0003754	mf: chaperon activity
Modulator protein function	GO:0003757	mf: chaperon activity
Modulator protein function	GO:0003758	mf: chaperon activity
Modulator protein function	GO:0003760	mf: chaperon activity
Modulator protein function	GO:0003761	mf: chaperon activity
Modulator protein function	GO:0003767	mf: co-chaperon activity
Modulator protein function	GO:0003768	mf: co-chaperon activity
Modulator protein function	GO:0003769	mf: co-chaperon activity
Modulator protein function	GO:0003770	mf: co-chaperon activity
Modulator protein function	GO:0003771	mf: co-chaperon activity
Modulator protein function	GO:0016238	bp: chaperone-related autophagy
Modulator protein function	GO:0007022	bp: chaperone-mediated tubulin folding
Modulator protein function	GO:0007023	bp: post-chaperonin tubulin folding pathway
Modulator protein function	GO:0006462	bp: protein complex assembly, multichaperone pathway
Modulator protein function	GO:0016465	cc:chaperonin ATPase complex
Modulator protein function	GO:0005832	cc: chaperonin-containing T-complex

Receptor	GO:0004872	mf: receptor activity
Receptor	GO:0005102	mf: receptor binding
Receptor	GO:0007166	bp: cell surface receptor linked signal transduction
Receptor	GO:0005057	mf: receptor signaling protein activity

Transcription factor	GO:0003700	mf: transcription factor activity
Transcription factor	GO:0000130	mf: transcription factor activity
Transcription factor	GO:0005667	cc: transcription factor complex
Transcription factor	GO:0042990	bp: regulation of transcription factor-nucleus import
Transcription factor	GO:0042991	bp: transcription factor-nucleus import

Intracellular matrix component	GO:0005622	cc: intracellular
Intracellular matrix component	GO:0046907	bp: intracellular transport
Intracellular matrix component	GO:0008092	mf: cytoskeletal protein binding

Extracellular matrix component	GO:0007160	bp: cell-matrix adhesion
Extracellular matrix component	GO:0009989	bp: cell-matrix recognition
Extracellular matrix component	GO:0005578	cc: extracellular matrix
Extracellular matrix component	GO:0005921	cc: gap junction
Extracellular matrix component	GO:0030055	cc: cell-matrix junction
Extracellular matrix component	GO:0005201	mf: extracellular matrix structural constituent
Extracellular matrix component	GO:0050840	mf: extracellular matrix binding
Transmembrane transporter	GO:0005215	mf: transporter activity
Transmembrane transporter	GO:0000036	mf: acyl carrier activity
Transmembrane transporter	GO:0019793	mf: ISG15 carrier activity

Channel	GO:0015267	mf: channel/pore class transporter activity
Channel	GO:0008282	cc: ATP-sensitive potassium channel complex
Channel	GO:0005891	cc: voltage-gated calcium channel complex
Channel	GO:0016935	cc: glycine-gated chloride channel complex
Channel	GO:0019183	cc: histamine-gated chloride channel complex
Channel	GO:0005892	cc: nicotinic acetylcholine-gated receptor-channel complex
Channel	GO:0008076	cc: voltage-gated potassium channel complex
Channel	GO:0001518	cc: voltage-gated sodium channel complex

Hormone	GO:0042562	mf: hormone binding
Hormone	GO:0005179	mf: hormone activity
Hormone	GO:0005131	mf: growth hormone receptor binding
Hormone	GO:0046879	bp: hormone secretion
Hormone	GO:0009725	bp: response to hormone stimulus
Hormone	GO:0009755	bp: hormone mediated signaling
Hormone	GO:0005831	cc: steroid hormone aporeceptor complex
Hormone	GO:0016914	cc: follicle-stimulating hormone complex

Immunoglobulin	GO:0019865	mf: immunoglobulin binding
Immunoglobulin	GO:0019763	mf: immunoglobulin receptor activity
Immunoglobulin	GO:0048305	bp: IG secretion
Immunoglobulin	GO:0045190	bp: isotype switching
Immunoglobulin	GO:0019814	cc: immunoglobulin complex

Cell signalling	GO:0019955	mf: cytokine binding
Cell signalling	GO:0005125	mf: cytokine activity
Cell signalling	GO:0019221	bp: cytokine and chemokine mediated signaling pathway
Cell signalling	GO:0042089	cytokine biosynthesis
Cell signalling	GO:0019838	mf: growth factor binding
Cell signalling	GO:0008083	mf: growth factor activity

**Table 2:** Comparison of GO mapping classification to Valle's categories

**Valle's Category**	**Original curation HDG count**	**True Positives**	**False Negatives**	**False Positives**	**GO-Mapping precision**	**GO-Mapping recall**
**Cell Signalling**	20	11	9	20	35%	55%
**Channel**	32	25	7	15	61%	78%
**Enzyme**	232	208	24	107	66%	90%
**Extracellular matrix component**	54	41	13	25	62%	76%
**Hormone**	14	14	0	1	93%	100%
**Immunoglobulin**	4	1	3	2	33%	25%
**Intracellular matrix component**	50	23	27	207	10%	46%
**Modulator of protein function**	105	4	101	5	44%	4%
**Receptor**	86	82	4	96	46%	95%
**Transcription factor**	79	62	17	10	85%	78%
**Transmembrane Transporter**	35	42	3	102	29%	93%

**Totals & overall accuracy scores**	**711**	**513**	**208**	590*	47%**	71%**

* Due to the possible assignment of more than one GO term per gene, we overestimated the overall FP rate as one gene classified multiple times due to multiple GO annotations lead to counting as a FP the same gene more than once.

** Overall accuracy scores are calculated from the overall True positive, false positive and false negative scores (they are not an average of the categorical accuracy scores)

**Table 3:** Comparison of GO clustering classification to Valle's categories

**Valle's Category**	**Original curation HDG count**	**True Positives**	**False Negatives**	**False Positives**	**GO-clustering precision**	**GO-clustering recall**
**Cell Signaling**	20	0	20	0	N/A	0%
**Channel**	32	28	4	7	34%	88%
**Enzyme**	232	143	89	15	85%	61%
**Extracellular matrix component**	54	17	37	21	63%	31%
**Hormone**	14	0	14	0	N/A	0%
**Immunoglobulin **	4	0	4	0	N/A	0%
**Intracellular matrix component**	50	42	8	10	43%	84%
**Modulator of protein function**	105	28	77	231	25%	27%
**Receptor**	86	58	28	10	73%	67%
**Transcription factor **	79	68	11	4	73%	86%
**Transmembrane Transporter**	35	15	20	42	32%	43%

**Totals & overall accuracy scores**	**711**	**399**	**312**	**340**	62%**	56%**

** Overall accuracy scores are calculated from the overall True positive, false positive and false negative scores (they are not an average of the categorical accuracy scores)

N/A: not applicable because precision cannot be calculated for categories with 0 true positive results and 0 false positives (zero divided by zero).

**Table 4:** Correlations of 14 clusters generated by the GO clustering method and Valle's categories of Human Disease Genes

		**GO-Clusters (Information Theoretic Distance)**														
	**Valle's Protein Function Categories**	**1**	**2**	**3**	**4**	**5**	**6**	**7**	**8**	**9**	**10**	**11**	**12**	**13**	**14**	**Total Genes in Valle's Category**
1	**Unknown**	3	4	2	8	4	2	1		1	8	2		2	2	**39**
2	**Enzyme**	4	10		4	5	2	2	**48**	**55**	7	**48**	1	**40**	6	**232**
3	**Transcription factor**	1	7	**45**	**23**		2			1						**79**
4	**Receptor**	7	11	2	1		**58**	1				6				**86**
5	**Hormone**		11				1	2								**14**
6	**Channel**	**28**				4										**32**
7	**Trans-membrane Transporter**	19				**15**				1						**35**
8	**EC Transport**	1	1			3				1		1			3	**10**
9	**Modulator of protein function**	5	16	4	17	8	6	3		8	17	7		2	**12**	**105**
10	**Other**	4	2		5	5	3				3	1		1	3	**27**
11	**Extracellular matrix component**	8	5	1		2	2	**17**			2		14		3	**54**
12	**Intracellular matrix component**	3	1			1	2				**23**		**19**		1	**50**
13	**Immunoglobulin**		1								2				1	**4**
14	**Cell Signaling**		**12**	2	1		1	1		1			1		1	**20**

Mapping of 14 clusters to 14 of Valle's classifications of HDGs. Numbers in the table denote the count of HDGs in each category. By design, multiple clusters could map to a protein function category, but each cluster could not be mapped to more than one category. The bold underlined numbers represent the true positive HDG and the selected Valle Category chosen for each GO Cluster. Other numbers in the cluster are considered as false positive in the evaluation. Valle's categories "unknown" and "others" were not evaluated because of their ambiguity.
